# Impact of Mutations in Highly Conserved Amino Acids of the HIV-1 Gag-p24 and Env-gp120 Proteins on Viral Replication in Different Genetic Backgrounds

**DOI:** 10.1371/journal.pone.0094240

**Published:** 2014-04-08

**Authors:** Yi Liu, Ushnal Rao, Jan McClure, Philip Konopa, Siriphan Manocheewa, Moon Kim, Lennie Chen, Ryan M. Troyer, Denis M. Tebit, Sarah Holte, Eric J. Arts, James I. Mullins

**Affiliations:** 1 Department of Microbiology, University of Washington School of Medicine, Seattle, Washington, United States of America; 2 Department of Medicine, University of Washington School of Medicine, Seattle, Washington, United States of America; 3 Department of Laboratory Medicine, University of Washington School of Medicine, Seattle, Washington, United States of America; 4 Division of Infectious Diseases, Department of Medicine, Case Western Reserve University, Cleveland, Ohio, United States of America; 5 Program in Biostatistics and Biomathematics, Fred Hutchinson Cancer Research Center, Seattle, Washington, United States of America; Centro de Biología Molecular Severo Ochoa (CSIC-UAM), Spain

## Abstract

It has been hypothesized that a single mutation at a highly conserved amino acid site (HCS) can be severely deleterious to HIV in most if not all isolate-specific genetic backgrounds. Consequently, potentially universal HIV-1 vaccines exclusively targeting highly conserved regions of the viral proteome have been proposed. To test this hypothesis, we examined the impact of 10 Gag-p24 and 9 Env-gp120 HCS single mutations on viral fitness. In the original founder sequence of the subject in whom these mutations were identified, all Gag-p24 HCS mutations significantly reduced viral replication fitness, including 7 that were lethal. Similar results were obtained at 9/10 sites when the same mutations were introduced into the founder sequences of two epidemiologically unlinked subjects. In contrast, none of the 9 Env-gp120 HCS mutations were lethal in the original founder sequence, and four had no fitness cost. Hence, HCS mutations in Gag-p24 are likely to be severely deleterious in different HIV-1 subtype B backgrounds; however, some HCS mutations in both Gag-p24 and Env-gp120 fragments can be well tolerated. Therefore, when designing HIV-1 immunogens that are intended to force the virus to nonviable escape pathways, the fitness constraints on the HIV segments included should be considered beyond their conservation level.

## Introduction

VaxGen's AIDSVAX vaccine, aimed at inducing neutralizing antibody responses [1]–[3], and Merck's rAd5-based T-cell vaccine [4] both failed to provide protection against HIV acquisition or control after HIV infection. The Merck vaccine, however, exerted selective pressure on breakthrough viruses [5] although no clear associations with specific immune responses have been identified [6]. Although the RV144 trial of vaccination with a combination of ALVAC and AIDSVAX showed a modest reduction in HIV-1 acquisition in Thailand [7], new vaccine approaches are clearly needed, especially those that target features of the virus that will elicit more potent protective immunity.

HIV-1's high mutation and recombination rates result in a structural plasticity that helps the virus to escape specific host immune responses while maintaining viral function. We and others [8]–[10] have proposed designing universal HIV-1 vaccine immunogens that would target highly conserved regions of the viral proteome in which mutations are predicted to compromise virus viability, and some would also omit viral sequences that may be preferentially recognized but afford little or no containment of the virus [8]. The underlying hypothesis of these approaches is that mutations at highly conserved amino acid sites (HCS) are likely to be detrimental to viral fitness and that the same HCS mutations will have a similar impact on different viral strains.

The degrees of variability of amino acid sites in HIV proteome sequences found in the Los Alamos HIV Sequence Database (HIVDB) have been associated with fitness constraints, i.e., amino acid sites with low variability might be under relatively stronger viral structural or functional constraints [11]–[14]. Fitness costs of escape mutations in Gag CTL epitopes have been reported (escape mutations underlined: TW10 (Gag 240–249, TSTLQEQIGW), KF11 (Gag 162–172, KAFSPEVIPMF), KK10 (Gag 163–172, KRWILLGLNK) and EW10 (Gag 203–212, ETINEEAAEW)) [14]–[18] involving relatively conserved amino acid sites (present in 88.8–97.7% of HIV-1 group M sequences in the HIVDB).

To test hypothesis that HCS mutations would be deleterious, we examined the impact of changing highly conserved amino acids in the HIV-1 Gag-p24 and Env-gp120 proteins. Amino acid sites over 98% conserved in HIV-1 group M in the HIVDB [8] were defined as HCS and others as variable sites (VS). We observed 10 Gag-p24 and 9 Env-gp120 HCS mutations in the sequences obtained from an antiretroviral therapy naïve subject during the first 4 years of HIV-1 subtype B infection. The impact of 2 Gag-p24 and 7 Env-gp120 HCS mutations in the subject's founder sequence were previously reported to be non-lethal [19]. Here, we determined the fitness impact of the remaining 10 HCS mutations, and summarize the results from study of the full 19 HCS mutations. To test the hypothesis of similar fitness impact of HCS mutations in different viral backgrounds, we examined the impact of the same 10 Gag-p24 HCS mutations in the founder viruses from two other HIV-1 subtype B infected subjects.

## Materials and Methods

**Figure 1:**
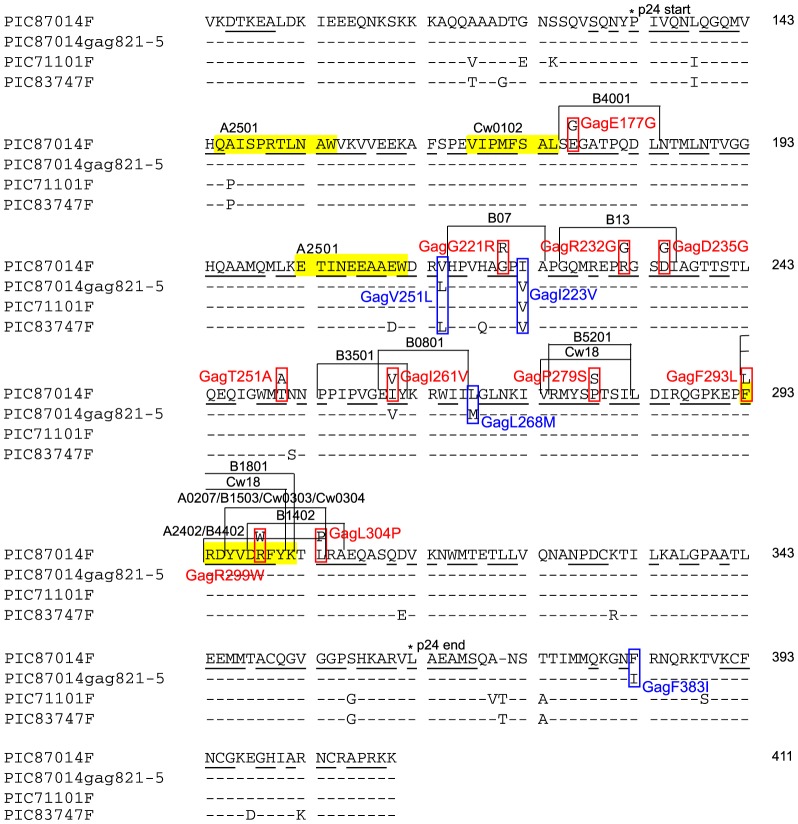
Gag-p24 founder sequences and HCS and VS mutations. Gag-p24 sequences (HIV-1_HXB2_ Gag 94–411) from PIC87014 (F, founder sequence from day 8 DPS and 821-5, sequence of clone 5 from 821 DPS), and founder sequences from PIC71101 (7 DPS) and PIC83747 (6 DPS) are shown. All sequences were obtained from previous studies [17], [20]. Numbers in the right column correspond to amino acid positions in HIV-1_HXB2_ Gag. * indicates the start and end of p24. All HCS are underlined, and the examined HCS and VS mutations are in red and blue boxes, respectively. The yellow highlights represent the CTL epitopes recognized by PIC87014, with their HLA restricting elements indicated (HLA alleles of PIC87014 are A*0201, A*2501, B*1801, B*5101, Cw*0102, and Cw*1203). The bracketed regions define the boundaries of optimally defined epitopes containing the HCS examined here, with the HLA restricting elements indicated. The sequences of the following optimally defined epitopes were different from the founder sequence of PIC87014: B*3501 restricted PPIPVGDIY (Gag 254–262), Cw*18 restricted VRMYSPVSI (Gag 274–282), A*2402 restricted RDYVDRFFKTL (Gag 294–304) and B*1503/Cw*0303/Cw*0304 restricted YVDRFFKTL (Gag 296–304), the underlined letters indicating the differences.

**Figure 2:**
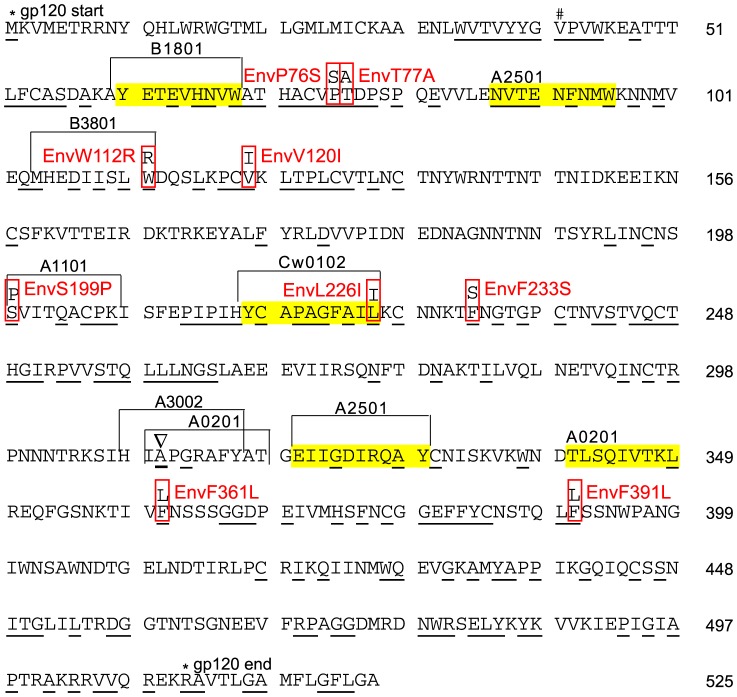
Env-gp120 founder sequence and HCS mutations of PIC87014. The Env founder sequence (HIV-1_HXB2_ Env 1–525) of PIC87014 is shown. All sequences were obtained from previous studies [17], [20]. # indicates the start of the sequence that was incorporated into the NL4-3 backbone. The letter marked with a ∇represents the HCS (Env 312_HXB2_) in the founder sequence (alanine) that is different from the group M or subtype B consensus (glycine). The sequences of the following optimally defined epitopes were different from the founder sequence: A*0202 restricted RGPGRAFVTI (311–320) and A*3002 restricted HIGPGRAFY (Env 310–318). See legend to Figure 1 for other conventions.

Identification of HCS mutations in Gag-p24 and Env-gp120. We previously obtained clonal viral sequences of *gag* encompassing the p24 coding region, and *env* encompassing nearly the complete gp120 coding region, from plasma (16 and 14 time points for *gag* and *env*, respectively, 12 sequences per time point on average) and PBMC (3 and 4 time points for *gag* and *env*, respectively, 6 sequences per time point on average) from an HIV-1 subtype B infected and antiretroviral treatment naive subject, PIC87014 (PIC1362 in the previous studies) [17], [20]. The subject was enrolled in the Seattle Primary Infection Clinic (PIC) cohort and followed for over four years beginning in acute infection [20]. Mutations in *gag* and *env* sequences at HCS were identified by comparison to the founder sequence (Figure 1 and 2). The founder sequence refers to the consensus of plasma viral sequences from the earliest time point sampled, in this case, 8 days post onset of acute symptoms of viral infection (DPS) [20], or ∼3 weeks post exposure to the virus [21]. The frequency of an amino acid at a certain position in HIV-1 group M or subtype B sequences in the HIVDB, as well as the group M or subtype B consensus sequences, were determined using alignments from [8].

Generation of recombinant plasmids containing founder, autologous or mutant viral sequences in the pNL4-3 backbone. Recombinant plasmids containing either the *gag*-p24 (HIV-1_HXB2_ nucleotides 1089–2022) or *env*-gp120 (nucleotides 6347–7802) founder sequence from PIC87014 in both pNL4-3VifA and pNL4-3VifB [14], [17], [20] were used in the current study. pNL4-3VifA corresponds to a plasmid with the NL4-3 sequence and pNL4-3VifB differs only by six synonymous nucleotide mutations introduced into the *vif* gene [14], [17]. These mutations had no detectable fitness impact and were used to facilitate differential recognition in competition assays [14], [17], [19]. In the current study, recombinant plasmids with PBMC-derived autologous *gag*-p24 sequences of PIC87014 from different time points in the backbone of pNL4-3VifA were generated using techniques described previously [17].

Recombinant plasmids containing *gag*-p24 founder sequences from two other HIV-1 subtype B infected subjects from the PIC cohort, PIC71101 and PIC83747, were also generated in the backbones of both pNL4-3VifA and pNL4-3VifB. These two subjects were selected because their HIV-1 infections were epidemiologically unlinked to PIC87014 and to each other. In addition, clonal sequences of nearly full length HIV-1 were obtained from their plasma samples collected during acute infection (7 DPS for PIC71101 and 6 DPS for PIC83747) in a previously study [22], from which we were able to identify clones with *gag* sequences identical to the founders. We PCR-amplified the founder *gag-p24* fragments (nucleotides 1089–2023), using primers GAD5F_YLlong (CATCAAAGGATAGATGTAAAAGACACCAAGGAAGC, HXB2 nucleotides 1054–1088) and GAD4R_YLlong (GTGTCCTTCCTTTCCACATTTCCAACAGC, nucleotides 2024–2052). Using the resulting PCR products as megaprimers [23]–[25] and pNL4-3VifA or pNL4-3VifB as template, we replaced the *gag* sequences of pNL4-3 with the amplified *gag* founder sequences from the two subjects. The magaprimer PCR reactions contained 0.2 mM dNTPs, 2U Phusion Hot Start High-Fidelity DNA polymerase (Finnzymes, Espoo, Finland), 500 ng of megaprimers and 50 ng of template in a final volume of 50 μl. The cycling conditions were 98°C for 30 sec, 35 cycles of 98°C for 10 sec, 48°C for 1 min and 72°C for 10 min, followed by 72°C for 10 min.

Single nucleotide changes were introduced into the founder sequences in pNL4-3VifA using the QuikChange II XL site-directed mutagenesis kit (Agilent, La Jolla, CA). The entire HIV-1 genome from each recombinant plasmid was sequenced to confirm the presence of the desired mutations and the absence of additional mutations.

Generation and testing of chimeric viruses. HEK293T cells [26] were transfected with recombinant plasmids using the FuGENE6 Transfection Reagent (Roche, San Francisco, CA) and cell-free supernatants were collected and used as viral stocks. The *gag* and *env* regions were then bulk sequenced to confirm the maintenance of the desired mutations. p24 production in transfection or culture supernatants was determined using an in-house double-antibody sandwich ELISA specific to the HIV-1 p24 antigen [27]. Viral infectivity was determined by 50% tissue culture infective dose (TCID_50_) [28] in PBMC (all from a single HIV-negative donor) using the p24 antigen capture assay.

Viral competitions. Viral competitions were performed as previously described [19]. Briefly, chimeric mutant viruses in NL4-3VifA backbones were competed against chimeric viruses with isogenic (founder) viral sequences in NL4-3VifB backbones. As a control, chimeric founder viruses in NL4-3VifA and NL4-3VifB backbones were competed against each other. The two competing viruses were mixed at a ratio of 1∶1 based on TCID_50_, and added to PBMC at a multiplicity of infection (MOI) of 0.005. Viruses with TCID_50_<200 IU/ml resulted in MOI<0.0002 (500 μl of viral mixture in 5×10^5^ PBMC), led to unreliable results [29] and were excluded from competition assays. Parallel monoinfections for each virus were conducted at a MOI of 0.005 to determine growth kinetics. Culture supernatants were collected daily from 2 to 9 days post infection. Sequences of the mutations of interest (*gag* or *env*) were assessed by bulk sequencing from culture supernatants at day 9. Virus production was also assessed in culture supernatants by real-time PCR using a common sense primer and probe, and *vifA* and *vifB*-specific antisense primers [19]. Chromatogram peak heights from sequencing reactions and real-time PCR results were compared for each competition, and found to provide consistent virus ratios. Therefore, recombination between the *vif* tag and mutation sites did not affect the observed virus ratios.

A mathematical model adopted from Wu et al [19], [30], [31] (http://indra.mullins.microbiol.washington.edu/grc/) was used to estimate the replication fitness cost of a mutation in the founder virus. Using a linear regression model, the net growth rates (*g*) of the mutant and the corresponding *vifB* founder virus was determined in competitions, as well as the growth rate difference (*d*) between the two, using the production of the two competing viruses during the period of exponential viral growth, from days 2 to 5 for *gag* and days 3 to 7 for *env* chimeric viruses.

## Results

Gag and Env founder sequences and HCS mutations. The *gag* and *env* founder sequences examined in this study encoded HIV-1_HXB2_ Gag residues 94–411 and Env residues 42–525. The three different *gag* founders had 11 to 15 amino acid differences (Figure 1), consistent with their HIV infections being epidemiologically unlinked. Sixty-two percent (*n* = 197) of the Gag and 36% (*n* = 175) of the Env sites corresponded to HCS (conserved in over 98% of known HIV-1 M group viral sequences). HCS in the founders were identical to the HIVDB group M consensus (Figure 1 and 2), except that the founder in PIC87014 had an alanine (A, marked with a∇ in Figure 2) at Env312 instead of the group M and subtype B consensus glycine (G).

Ten mutations were observed at HCS in Gag (all in p24) and 9 in Env (all in gp120) over the first 4 years of infection in PIC87014 (Figure 1, 2 and Table 1), all involving single nucleotide changes. Except for EnvL226I, which was later fixed in the subject [20], the other 18 HCS mutations were rare - observed only once in PBMC-derived sequences and nearly absent in plasma-derived sequences (Table 1).

**Table 1:** Founder and mutant amino acids of the HCS and VS mutations in the HIVDB and PIC87014.

	Mutation	Founder % (M, B)^a^	Mutant % (M, B)^a^	Mutant presence in PIC87014	
				PBMC^b^	Plasma^b^
HCS	GagE177G	E (98.6, 100)	G (0, 0)	1/21	5/200^c^
	GagG221R	G (99.4, 97.5)	R (0.16, 0)	1/21	0/200
	GagR232G	R (98.7, 96.9)	G (0.32, 0)	1/21	0/200
	GagD235G	D (99.3, 97.9)	G (0, 0)	1/21	0/200
	GagT251A	T (99.4, 98)	A (0.16, 0)	1/21	0/200
	GagI261V	I (99.5, 98)	V (0, 0)	1/21	0/200
	GagP279S	P (99.4, 97.5)	S (0.32, 0.5)	1/21	0/200
	GagF293L	F (99.5, 98)	L (0, 0)	1/21	0/200
	GagR299W	R (99, 98)	W (0.16, 0)	1/21	0/200
	GagL304P	L (99.2, 98)	P (0, 0)	1/21	0/200
	EnvP76S	P (100, 100)	S (0, 0)	1/23	0/155
	EnvT77A	T (98.9, 97.5)	A (0.34, 0)	1/23	1/155
	EnvW112R	W (99.9, 99.5)	R (0, 0)	1/23	1/155
	EnvV120I	V (98.6, 100)	I (0.8, 0)	1/23	0/155
	EnvS199P	S (99, 100)	P (0.11, 0)	1/23	1/155
	EnvF233S	F (98.9, 99.5)	S (0.12, 0)	1/23	2/155^c^
	EnvF361L	F (99.2, 99)	L (0.47, 0.51)	1/23	0/155
	EnvF391L	F (99.2, 99)	L (0.12, 0)	1/23	0/155
	EnvL226I	L (98.3, 95.9)	I (1.2, 3)	18/23	83/155^c^
VS	GagV215L	V (16, 12)	L (73, 81)	11/21	See Figure 3F
	GagI223V	I (63, 70)	V (27, 22)	7/21	See Figure 3F
	GagL268M	L (93, 83)	M (5, 13)	6/21	See Figure 3F
	GagF383I	F (97, 92)	I (0.5, 0.5)	2/21	See Figure 3F

a The database frequencies of the founder or mutant amino acids in HIV-1 group M (M) and subtype B (B) sequences in the HIVDB, based on alignments from [8].

b From PBMC, a total of 21 gag-p24 sequences from 3 time points and 23 env-gp120 sequences form 4 time points were obtained. From plasma, a total of 200 *gag-p24* sequences from 16 time points and 155 *env-gp120* sequences form 14 time points were obtained [14], [20], [34].

c GagE177G was observed in 5 sequences obtained from 4 time points. EnvF233S was observed in 2 sequences obtained from 2 time points. EnvL226I was fixed (100% in sequenced plasma viral genomes) since 344 DPS [14], [20], [34].

**Figure 3:**
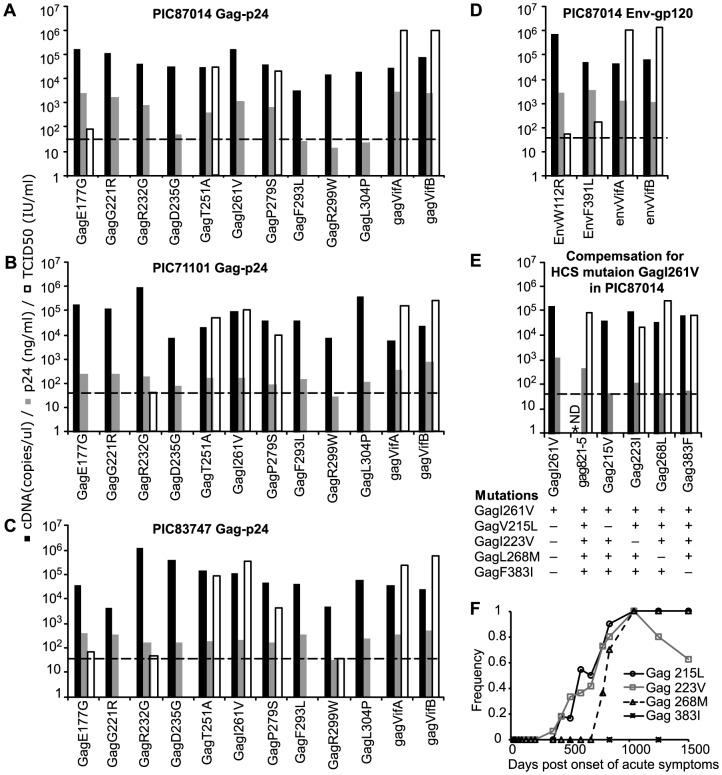
Impact of Gag-p24 and Env-gp120 HCS mutations on viral growth and compensation by VS mutations. cDNA copy numbers (black bars), p24 levels (gray bars) and TCID_50_ (open bars) derived from transfection supernatants of the *gag* founders and mutants of PIC87014 (A), PIC71101 (B) and PIC83747 (C), as well as the *env* founder and mutants of PIC87014 (D). Mutations that resulted in undetectable TCID_50_ (<38 IU/ml, the dotted horizontal lines represent this threshold) were considered lethal. E) Compensatory impact of VS mutations that were linked to GagI261V. Gag215V, Gag223I, Gag268L and Gag383F are mutants of PIC87014gag821-5 with individual VS mutations reverted to the PIC87014 founder state. The +/− sign underneath each virus represents the presence/absence of the indicated mutation. ND, not determined. F) Frequencies of the VS mutations in the plasma-derived viral sequences of PIC87014 over time.

Lethality of Gag-p24 and Env-gp120 HCS mutations. The HCS mutation EnvL226I had previously been shown to have little impact on viral replication fitness in a virus encoding the autologous PIC87014 founder protein [17]. Two Gag-p24 and six Env-gp120 HCS mutations [GagT251A and GagP279S (gag1 and gag2 in [19]), EnvP76S, EnvT77A, EnvV120I, EnvS199P, EnvF233S and EnvF361L (env 1–6 in [19])] had also been reported to be non-lethal in the PIC87014 founder [19]. We introduced the remaining 10 HCS mutations individually into the *gag* or *env* founder sequences of PIC87014, and the same 10 Gag-p24 HCS mutations individually into the *gag* founder sequences of PIC71101 and PIC83747. The copy numbers of cDNA derived from all transfection supernatants were over 3.3x10^3^ copies/μl, indicating efficient transfection and virion formation (Figure 3).

All founder viruses (Figure 3A-D, lanes marked gagVifA or VifB and envVifA or VifB) had infectious titers (TCID_50_, open bars) over 10^5^ IU/ml. However, seven Gag-p24 HCS mutations were apparently lethal in the PIC87014 founder (TCID_50_<38 IU/ml), and one (GagE177G) led to very low TCID_50_ (<100 IU/ml, Figure 3A). Both Env-gp120 HCS mutants (EnvW112R and EnvF391L) were infectious, although with relatively low titers (TCID_50_<200 IU/ml, Figure 3D). Therefore, all 9 Env-gp120 HCS mutations were non-lethal. In the original founder background then, HCS mutations in Gag-p24 were more likely to be lethal than those in Env-gp120 (*p* = 0.003, 7/10 vs 0/9, Fisher's exact test).

The impact of 9/10 Gag-p24 HCS mutations on viral growth was consistent in the PIC71101 and PIC83747 founder backgrounds (Figure 3A, B and C). Of the seven lethal mutations within the PIC87014 founder, 4 (GagG221R, GagD235G, GagF293L and GagL304P) were also lethal in both heterologous founder backgrounds, 2 others (GagR299W and GagR232G) were lethal or resulted in barely infectious viruses (TCID_50_ ≤50 IU/ml), and only 1 (GagI261V) failed to diminish virus infectivity significantly, with TCID_50_ levels 0.4 to 0.8 log_10_ lower than those of the corresponding founder viruses (Figure 3B and C).

VS mutations that compensate for lethal/deleterious HCS mutations. The strongly deleterious or lethal impact of the Gag-p24 HCS mutations that we observed might be compensated for by linked VS mutations. We therefore examined chimeric viruses containing PBMC-derived PIC87014 *gag* (from 298, 487 and 821 DPS, Figure S1) that had at least one of the lethal Gag-p24 HCS mutations. Chimera PIC87014gag821-5, containing a *gag* sequence from 821 DPS, had a TCID_50_ that was only 1 log_10_ lower than the founder (Figure 3E), whereas the other chimeras were defective (data not shown). PIC87014gag821-5 had the lethal HCS mutation GagI261V along with four VS mutations (Figure 1). Except for GagF383I, the other three VS mutations were in Gag-p24 and were abundant in HIV-1 group M and subtype B sequences in the HIVDB as well as in PIC87014 (Table 1 and Figure 3F). As noted above, mutation GagI261V in the other two *gag* founder backgrounds was not lethal – however, these founders shared GagI223V and PIC83747 also shared GagV215L (Figure 1).

To identify potential compensatory mutations, we reverted the four VS mutations individually in PIC87014gag821-5 to the founder amino acids. The mutant with the reversion to Gag215V was defective, whereas the other three reversion mutants had infectious titers comparable to that of chimera PIC87014gag821-5 (Figure 3E). Therefore, the VS mutation GagV215L was essential to compensate for the lethality of the HCS mutation GagI261V in PIC87014gag821-5 (and possibly in PIC83747).

**Figure 4:**
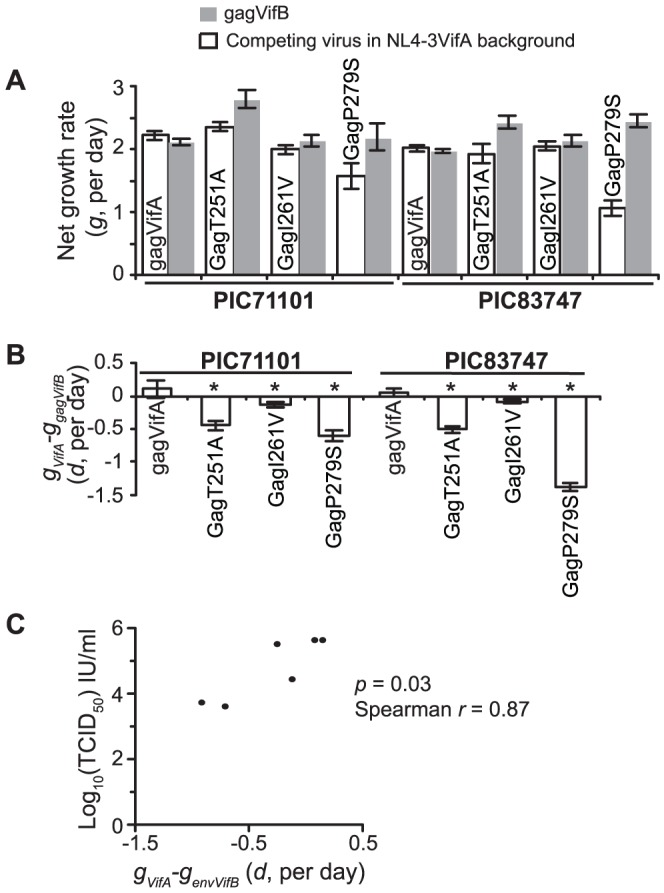
Impact of non-lethal HCS mutations on viral replication fitness. A) Net growth rates of individual *gag* viruses in competitions. B) Net growth rate differences between the mutants and *vifB* founder *gag* viruses in competitions. gagVifA and gagVifB have founder virus *gag*-p24 (HIV-1_HXB2_ nucleotides 1089–2022) inserted into the NL4-3 backbone and differ only by 6 synonymous site mutations in the *vif* gene. Mutant and founder viruses in the *vifA* backbone were competed against founder viruses in the *vifB* backbone. Reported are the means and 95% confidence intervals from competition experiments carried out in triplicate. * indicates significant differences (*p*<0.05, calculated using the method developed in [19]) between 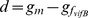 (net growth rate difference between the mutant and *vifB* founder viruses) and 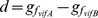 (net growth rate difference between *vifA* and *vifB* founders). A value of 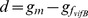 that was significantly smaller was interpreted as a fitness cost from the mutation; a value of 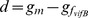 that was significantly greater was interpreted as a fitness gain; otherwise it was interpreted as no fitness impact. C) Correlation between viral replication fitness and TCID_50_ in Env-gp120 HCS mutants.

Impact of non-lethal HCS mutations on replication fitness. Consistent with their impact in the PIC87014 founder background [19], the GagT251A and GagP279S mutations significantly reduced viral replication fitness in PIC71101 and PIC83747 founder backgrounds (Figure 4A and B). Mutation GagI261V also significantly reduced viral replication fitness in the heterologous backgrounds. In contrast, our previous studies [17], [19] showed that only 3/7 Env-gp120 HCS mutations examined (EnvS199P, EnvF233S and EnvF361L) significantly reduced fitness, whereas the other 4 had little fitness impact (EnvP76S and EnvL226I) or slightly but significantly increased fitness (EnvT77A, EnvV120I) in the autologous PIC87014 background. Replication fitness of the Env-gp120 HCS mutants correlated with their TCID_50_ (*p* = 0.03, Spearman correlation, Figure 4C). Three Gag-p24 and Env-gp120 HCS mutants (GagE177G, EnvW112R and EnvF391L) could not be examined because of the low viral infectious titers, and hence they were likely to impose great fitness cost.

## Discussion

To test the hypothesis that HCS mutations are likely to be universally deleterious to HIV-1, we examined the impact on viral growth in cell culture and fitness of 10 Gag-p24 and 9 Env-gp120 HCS mutations found in an HIV-1 subtype B infected subject (PIC87014). When introduced into his founder sequence, 7 of the Gag-p24 HCS mutations were lethal and the other 3 significantly reduced viral replication fitness. In contrast, none of the Env-gp120 HCS mutations were lethal and only 5 significantly reduced fitness. When the 10 Gag-p24 HCS mutations were introduced into the founder sequences of two other HIV-1 subtype B infected subjects, similar fitness results were obtained except for one mutation, GagI261V, which was lethal in the PIC87014 founder but only slightly reduced viral fitness in the other virus backgrounds. The lethality of the GagI261V mutation in the PIC87014 founder could be compensated for by a VS mutation also found in the subject. Thus, most HCS mutations in Gag-p24 were lethal or debilitating, while many mutations at HCS within Env-gp120 were well tolerated in cell culture. The deleterious fitness impact of HCS Gag-p24 mutations was generally consistent among different subtype B viral backgrounds. However, at least in one case, compensation for a lethal HCS mutation was evident within the Gag-p24 protein.

Because HCS are over 98% conserved in the HIVDB, we expect that the appearance of HCS mutations in the viral population of an infected subject would generally be rare. This makes the study of naturally occurring HCS mutations difficult. Of the 19 HCS mutations in this study, only EnvL226I was observed with high frequency in PIC87014. Other HCS mutations were rarely found in the subject, with no evidence of hypermutation [32]. Although we did not rule out the possibility that these mutations were induced by PCR error, they all corresponded to single point mutations and thus represented potential mutational pathways. Their fitness impact helps us understand the viral tolerance to HCS mutations and the influence of different viral genetic backgrounds.

Although we did not examine every possible mutation involving the 10 Gag-p24 HCS, our results of consistently deleterious fitness impact of most of these mutations in different viral backgrounds support that HCS in Gag-p24 might largely result from structural or functional constraints. Indeed, 6/10 Gag-p24 HCS have been reported to play a critical role in HIV structure and function (Table 2). In addition, 9/10 Gag-p24 HCS mutations (all except mutation GagT251A) were in optimal CTL epitopes [33] and half were located in epitope regions restricted by multiple HLA alleles (Table 3). Except for EnvL226I, we did not examine the association of the other HCS mutations with CTL escape. However, these Gag-p24 HCS in optimal epitopes likely have experienced CTL pressure in the infected population during the HIV pandemic. Host immune pressure, especially CTL pressure, is a driving force for viral amino acid polymorphism within HIV infected subjects and in the infected human population [12], [20]. However, if amino acid sites under immune pressure are critical for viral function or structure, they will likely show high conservation. Therefore, for these Gag-p24 HCS, their location in optimal CTL epitopes further supports their tight fitness constraints. Two HCS mutations (GagF293L and GagR299W) were located within an epitope (FK10, Figure 1 and Table 3) recognized throughout the first 4 years of infection in PIC87014 [14], [20], [34]. Inability of the virus to find a viable escape pathway might be one reason that the CTL response persisted. Our results showed that at least two of the mutational pathways would have been lethal.

**Table 2:** Known roles of the examined HCS in HIV-1 structure and function.

Examined HCS	Role in viral structure and function	Reference
G221	Located in the cyclophilin A binding loop. A key determinant for cyclophilin A recognition.	[37]
R232	Located on the surface of HIV capsid. A double mutation of R232A/S234A impairs viral assembly and infectivity.	[38]
P279	Involved in the high affinity capsid dimer interface.	[39]
F293	Located in the major homology region (MHR^a^, Gag 285-304) of p24, which is important for viral assembly, maturation and infectivity. Contributes to the hydrophobic core of the capsid protein.	[38], [39]
R299	Located in MHR and involved in the hydrogen-binding network that stabilizes the MHR structure and links it to adjacent helices.	[39]
L304	Located in MHR and contributes to the hydrophobic core of the capsid protein.	[39]

a MHR is a sequence found in many retroviruses and in the yeast transposon Ty3 [40]-[42].

**Table 3:** HCS mutations located in known CTL^a^.

CTL epitopes	Gag-p24	Env-gp120
Recognized by PIC87014	GagF293L and GagR299W (FRDYVDRFYK^b^)	EnvL226I (YCAPAGFAIL b)
Not recognized by PIC87014	GagE177G, GagG221R, GagR232G, GagD235G, GagI261V, GagP279S and GagL304P (*n* = 7)	EnvW112R and EnvS199P (*n* = 2)
Restricted by multiple HLA alleles	GagI261V, GagP279S, GagF293L, GagR299W and GagL304P (*n* = 5)	None

a In the examined regions, 44 Gag and 17 Env CTL epitopes have been optimally defined [33], among which 4 Gag and 3 Env epitopes are known to be restricted by HLA alleles of PIC87014 (A*0201, A*2501, B*1801, B*5101, Cw*0102, and Cw*1203) and all 7 were recognized by the subject [20]. PIC87014 also recognized 2 additional Env-gp120 epitopes (NW9, Env 88–96 and TL9, Env 341–349, Figure 2).

b FRDYVDRFYK (FK10, Gag 293–302) is B*1801 restricted. YCAPAGFAIL (YL10, Env 271–226) is Cw*0102 restricted. The underlined letters indicate the location of the HCS mutations.

Mutations at 4/9 Env-gp120 HCS did not reduce replication fitness (e.g., EnvP76S, EnvT77A, EnvV120I and EnvL226I), indicating that these sites might not be critical for the virus. However, one possible reason for their high conservation would be a lack of selection pressure in the HIV infected population. In support of this hypothesis, we found that three of these sites (EnvP76S, EnvT77A and EnvV120I) were not located in CTL epitopes recognized by PIC8714, nor in optimally defined CTL epitopes [33]. They were also not in epitopes known to be recognized by human HIV antibodies (http://www.hiv.lanl.gov/content/immunology/maps/ab/gp160.html). However, it should be noted that the optimally defined CTL epitope list [33] is an under appreciation of CTL recognition, especially within Env-gp120. For example, 2 CTL epitopes in Env-gp120 recognized by PIC87014 were not included in the list, and of the minimum of 25 epitopes recognized by PIC87014, only 14 had been defined previously [20]. The list of known HIV antibody epitopes is also far from complete. Therefore, it is possible that these sites have experienced immune pressure as well but with only a small replication advantage of the founder amino acids at these sites, too small to be reliably detected by our assay, but enough to drive them to high conservation in the HIVDB. Finally, the fitness impact of mutations was examined in cell culture using the founder viral *gag-p24* or *env-gp120* gene fragments in the NL4-3 backbone, which might be different from the impact on autologous founder viruses in vivo. Hence, we might not be able to adequately recapitulate an in vivo condition in which the replication advantages of these HCS might be more evident.

Even some Env-gp120 HCS mutations that mediate immune escape could be well tolerated. HCS mutation EnvL226I (Figure 2 and Table 3) mediated CTL escape with little viral replication fitness cost [17] and became fixed within a year of infection in PIC87014 [20], [34]. Interestingly, at HCS Env312 (Figure 2, marked with a ∇), only alanine (but not group M or subtype B consensus of glycine) was observed in PIC87014 during the first four years of infection [20]. In the plasma obtained from his transmission partner [20] sampled ∼5 weeks following transmission, Env312A was observed in 8/10 and Env312G in 2/10 of the viral genomes. Env312 was located in two optimally defined CTL epitopes restricted by A*3002 or A*0201, the latter HLA allele being shared by PIC87014 and his partner [20]. These data suggest that Env312A might be involved in escape from A*0201-mediated CTL recognition (we were unable to determine CTL responses in the partner due to lack of PBMC). In addition, Env321A was most likely transmitted from the partner and remained stable in PIC87014. Hence, there was at most a small, if any, selective advantage for the virus to revert to the group M consensus of Env312G in PIC87014. Taken together, the tolerance of CTL escape mutations at some Env-gp120 HCS suggests that their high conservation might result from rare or generally ineffective immune responses.

Fitness-reducing drug resistance and CTL escape mutations in HIV can sometimes be restored by compensatory mutations [35], [36]. Here we showed that lethality of the HCS mutation GagI261V could also be rescued. The compensatory mutation GagV215L was shown previously to mediate CTL escape by impairing proper epitope processing of an adjacent epitope (EW10 (Gag 203–212, ETINEEAAEW) [17]) with little impact on viral replication fitness, thus permitting the reversion of the highly debilitating escape mutation (Gag E207D) within that epitope [14], [17].

In summary, our studies demonstrate that HCS in the Gag-p24 protein were likely to be involved in important structural and functional features of the viral protein, while mutations of HCS in the gp120 component of Env were not predictive of impaired viral growth. However, even some HCS mutations in Gag-p24 are non-lethal. Together with potential compensation from VS mutations, it is likely that there are viable mutational pathways for some highly conserved HIV segments. Therefore, for vaccines aiming to generate cellular immune responses targeting largely immutable protein segments [8], fitness constraints of the highly conserved HIV elements included in the immunogens have to be carefully assessed.

## Supporting Information

Figure S1 **PIC87014 Gag-p24 founder sequences and autologous sequences with lethal HCS mutations.** All sequences were obtained from previous studies [17], [20]. Numbers in the right column correspond to amino acid positions in HIV-1_HXB2_ Gag.
(EPS)
